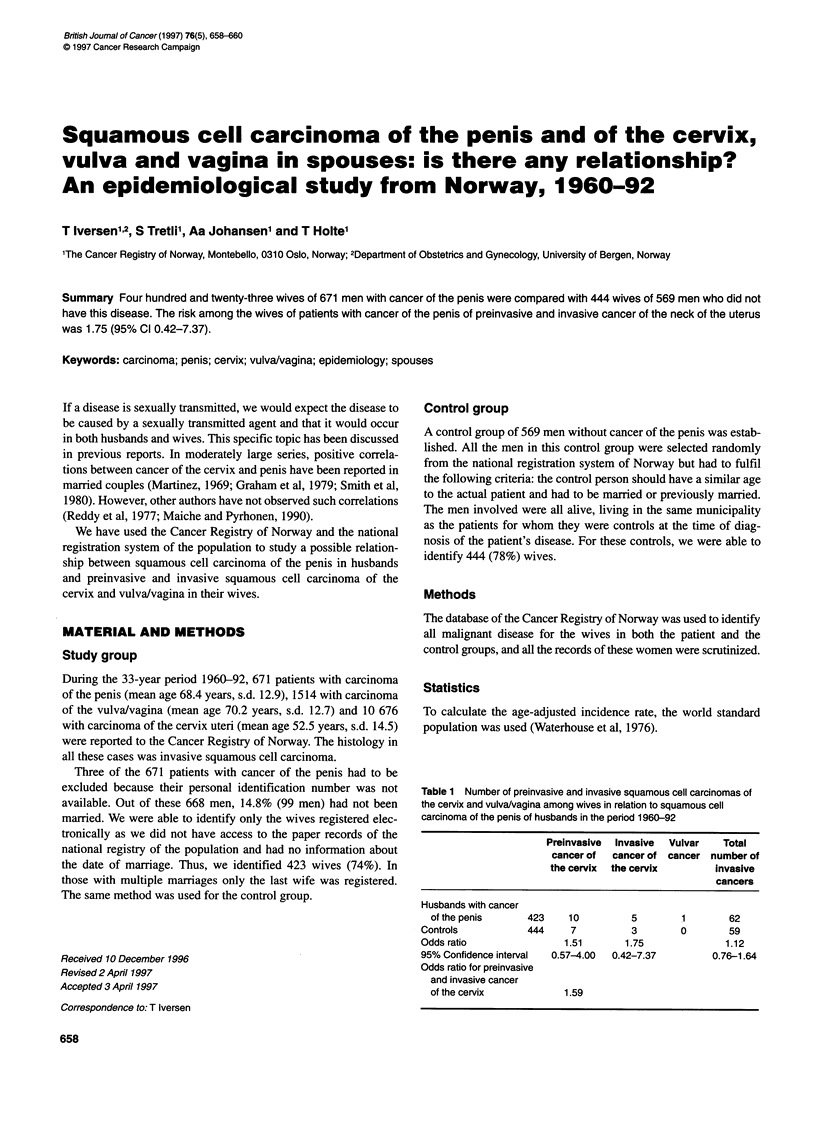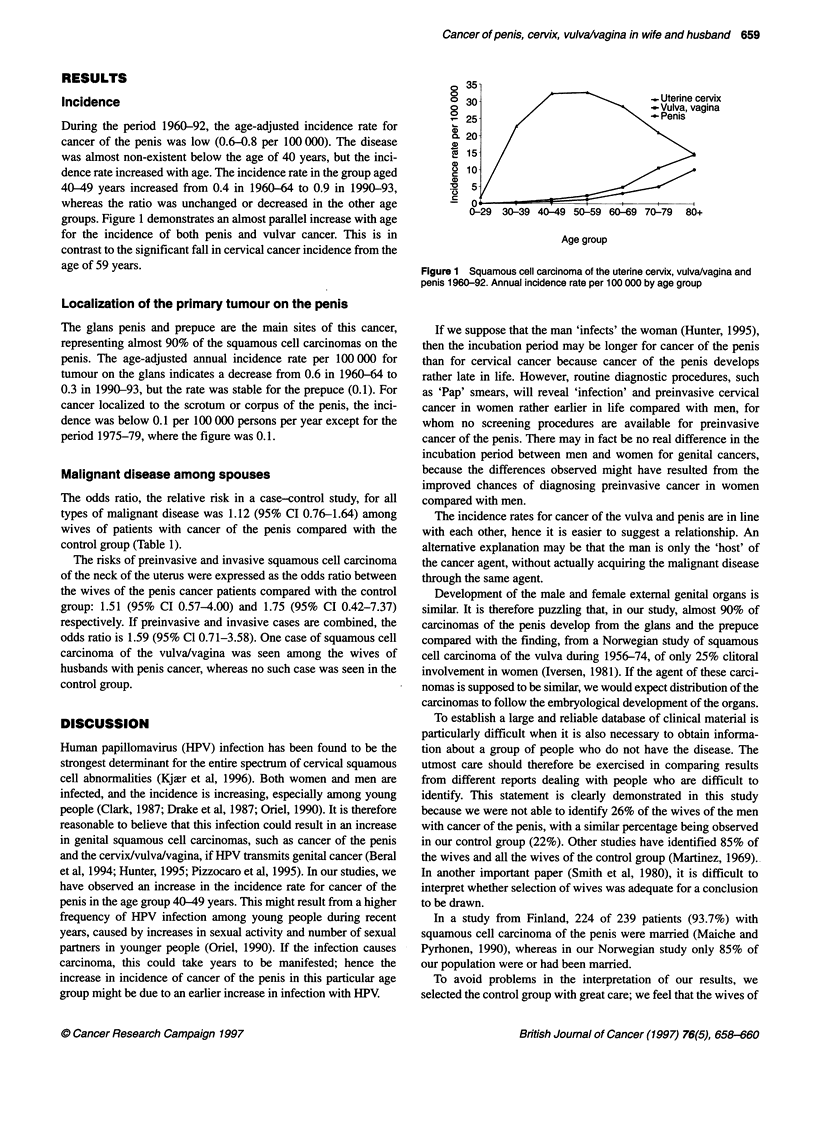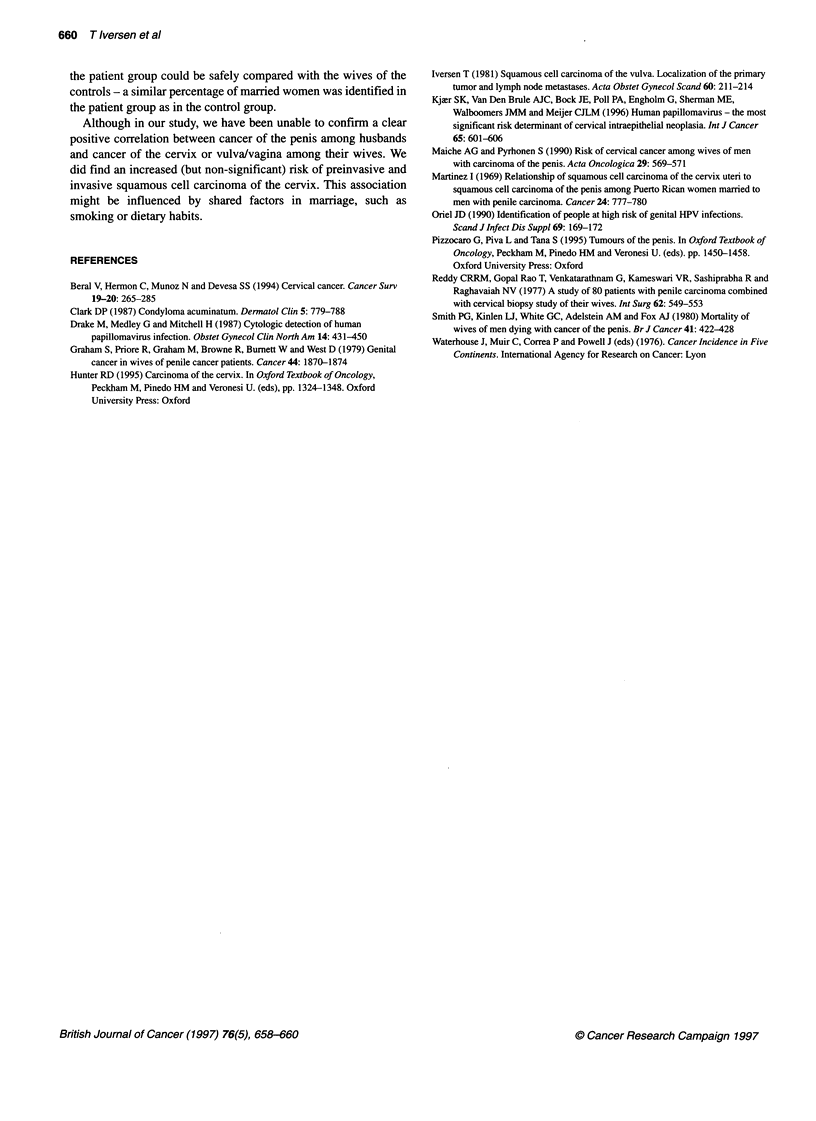# Squamous cell carcinoma of the penis and of the cervix, vulva and vagina in spouses: is there any relationship? An epidemiological study from Norway, 1960-92.

**DOI:** 10.1038/bjc.1997.441

**Published:** 1997

**Authors:** T. Iversen, S. Tretli, A. Johansen, T. Holte

**Affiliations:** The Cancer Registry of Norway, Montebello, Oslo.

## Abstract

Four hundred and twenty-three wives of 671 men with cancer of the penis were compared with 444 wives of 569 men who did not have this disease. The risk among the wives of patients with cancer of the penis of preinvasive and invasive cancer of the neck of the uterus was 1.75 (95% CI 0.42-7.37).


British Journal of Cancer (1997) 76(5), 658–660
© 1997 Cancer Research Campaign

### Squamous cell carcinoma of the penis and of the cervix, vulva and vagina in spouses: is there any relationship? An epidemiological study from Norway, 1960–92

**T Iversen[1,2], S Tretli[1], Aa Johansen[1] and T Holte[1]**

[1]The Cancer Registry of Norway, Montebello, 0310 Oslo, Norway; [2]Department of Obstetrics and Gynecology, University of Bergen, Norway

**Summary** Four hundred and twenty-three wives of 671 men with cancer of the penis were compared with 444 wives of 569 men who did not have this disease. The risk among the wives of patients with cancer of the penis of preinvasive and invasive cancer of the neck of the uterus was 1.75 (95% CI 0.42–7.37).

**Keywords:** carcinoma; penis; cervix; vulva/vagina; epidemiology; spouses

If a disease is sexually transmitted, we would expect the disease to be caused by a sexually transmitted agent and that it would occur in both husbands and wives. This specific topic has been discussed in previous reports. In moderately large series, positive correlations between cancer of the cervix and penis have been reported in married couples (Martinez, 1969; Graham et al, 1979; Smith et al, 1980). However, other authors have not observed such correlations (Reddy et al, 1977; Maiche and Pyrhonen, 1990).

We have used the Cancer Registry of Norway and the national registration system of the population to study a possible relationship between squamous cell carcinoma of the penis in husbands and preinvasive and invasive squamous cell carcinoma of the cervix and vulva/vagina in their wives.

### MATERIAL AND METHODS

### Study group

During the 33-year period 1960–92, 671 patients with carcinoma of the penis (mean age 68.4 years, s.d. 12.9), 1514 with carcinoma of the vulva/vagina (mean age 70.2 years, s.d. 12.7) and 10 676 with carcinoma of the cervix uteri (mean age 52.5 years, s.d. 14.5) were reported to the Cancer Registry of Norway. The histology in all these cases was invasive squamous cell carcinoma.

Three of the 671 patients with cancer of the penis had to be excluded because their personal identification number was not available. Out of these 668 men, 14.8% (99 men) had not been married. We were able to identify only the wives registered electronically as we did not have access to the paper records of the national registry of the population and had no information about the date of marriage. Thus, we identified 423 wives (74%). In those with multiple marriages only the last wife was registered. The same method was used for the control group.

*Received 10 December 1996*
*Revised 2 April 1997*
*Accepted 3 April 1997*

*Correspondence to:* T Iversen

### Control group

A control group of 569 men without cancer of the penis was established. All the men in this control group were selected randomly from the national registration system of Norway but had to fulfil the following criteria: the control person should have a similar age to the actual patient and had to be married or previously married. The men involved were all alive, living in the same municipality as the patients for whom they were controls at the time of diagnosis of the patient's disease. For these controls, we were able to identify 444 (78%) wives.

### Methods

The database of the Cancer Registry of Norway was used to identify all malignant disease for the wives in both the patient and the control groups, and all the records of these women were scrutinized.

### Statistics

To calculate the age-adjusted incidence rate, the world standard population was used (Waterhouse et al, 1976).

**Table 1** Number of preinvasive and invasive squamous cell carcinomas of the cervix and vulva/vagina among wives in relation to squamous cell carcinoma of the penis of husbands in the period 1960–92

| | | Preinvasive cancer of the cervix | Invasive cancer of the cervix | Vulvar cancer | Total number of invasive cancers |
|---|---|---|---|---|---|
| Husbands with cancer of the penis | 423 | 10 | 5 | 1 | 62 |
| Controls | 444 | 7 | 3 | 0 | 59 |
| Odds ratio | | 1.51 | 1.75 | | 1.12 |
| 95% Confidence interval | | 0.57–4.00 | 0.42–7.37 | | 0.76–1.64 |
| Odds ratio for preinvasive and invasive cancer of the cervix | | 1.59 | | | |

### RESULTS

### Incidence

During the period 1960–92, the age-adjusted incidence rate for cancer of the penis was low (0.6–0.8 per 100 000). The disease was almost non-existent below the age of 40 years, but the incidence rate increased with age. The incidence rate in the group aged 40–49 years increased from 0.4 in 1960–64 to 0.9 in 1990–93, whereas the ratio was unchanged or decreased in the other age groups. Figure 1 demonstrates an almost parallel increase with age for the incidence of both penis and vulvar cancer. This is in contrast to the significant fall in cervical cancer incidence from the age of 59 years.

**Figure 1** Squamous cell carcinoma of the uterine cervix, vulva/vagina and penis 1960–92. Annual incidence rate per 100 000 by age group

### Localization of the primary tumour on the penis

The glans penis and prepuce are the main sites of this cancer, representing almost 90% of the squamous cell carcinomas on the penis. The age-adjusted annual incidence rate per 100 000 for tumour on the glans indicates a decrease from 0.6 in 1960–64 to 0.3 in 1990–93, but the rate was stable for the prepuce (0.1). For cancer localized to the scrotum or corpus of the penis, the incidence was below 0.1 per 100 000 persons per year except for the period 1975–79, where the figure was 0.1.

### Malignant disease among spouses

The odds ratio, the relative risk in a case–control study, for all types of malignant disease was 1.12 (95% CI 0.76–1.64) among wives of patients with cancer of the penis compared with the control group (Table 1).

The risks of preinvasive and invasive squamous cell carcinoma of the neck of the uterus were expressed as the odds ratio between the wives of the penis cancer patients compared with the control group: 1.51 (95% CI 0.57–4.00) and 1.75 (95% CI 0.42–7.37) respectively. If preinvasive and invasive cases are combined, the odds ratio is 1.59 (95% Cl 0.71–3.58). One case of squamous cell carcinoma of the vulva/vagina was seen among the wives of husbands with penis cancer, whereas no such case was seen in the control group.

### DISCUSSION

Human papillomavirus (HPV) infection has been found to be the strongest determinant for the entire spectrum of cervical squamous cell abnormalities (Kjær et al, 1996). Both women and men are infected, and the incidence is increasing, especially among young people (Clark, 1987; Drake et al, 1987; Oriel, 1990). It is therefore reasonable to believe that this infection could result in an increase in genital squamous cell carcinomas, such as cancer of the penis and the cervix/vulva/vagina, if HPV transmits genital cancer (Beral et al, 1994; Hunter, 1995; Pizzocaro et al, 1995). In our studies, we have observed an increase in the incidence rate for cancer of the penis in the age group 40–49 years. This might result from a higher frequency of HPV infection among young people during recent years, caused by increases in sexual activity and number of sexual partners in younger people (Oriel, 1990). If the infection causes carcinoma, this could take years to be manifested; hence the increase in incidence of cancer of the penis in this particular age group might be due to an earlier increase in infection with HPV.

If we suppose that the man 'infects' the woman (Hunter, 1995), then the incubation period may be longer for cancer of the penis than for cervical cancer because cancer of the penis develops rather late in life. However, routine diagnostic procedures, such as 'Pap' smears, will reveal 'infection' and preinvasive cervical cancer in women rather earlier in life compared with men, for whom no screening procedures are available for preinvasive cancer of the penis. There may in fact be no real difference in the incubation period between men and women for genital cancers, because the differences observed might have resulted from the improved chances of diagnosing preinvasive cancer in women compared with men.

The incidence rates for cancer of the vulva and penis are in line with each other, hence it is easier to suggest a relationship. An alternative explanation may be that the man is only the 'host' of the cancer agent, without actually acquiring the malignant disease through the same agent.

Development of the male and female external genital organs is similar. It is therefore puzzling that, in our study, almost 90% of carcinomas of the penis develop from the glans and the prepuce compared with the finding, from a Norwegian study of squamous cell carcinoma of the vulva during 1956–74, of only 25% clitoral involvement in women (Iversen, 1981). If the agent of these carcinomas is supposed to be similar, we would expect distribution of the carcinomas to follow the embryological development of the organs.

To establish a large and reliable database of clinical material is particularly difficult when it is also necessary to obtain information about a group of people who do not have the disease. The utmost care should therefore be exercised in comparing results from different reports dealing with people who are difficult to identify. This statement is clearly demonstrated in this study because we were not able to identify 26% of the wives of the men with cancer of the penis, with a similar percentage being observed in our control group (22%). Other studies have identified 85% of the wives and all the wives of the control group (Martinez, 1969). In another important paper (Smith et al, 1980), it is difficult to interpret whether selection of wives was adequate for a conclusion to be drawn.

In a study from Finland, 224 of 239 patients (93.7%) with squamous cell carcinoma of the penis were married (Maiche and Pyrhonen, 1990), whereas in our Norwegian study only 85% of our population were or had been married.

To avoid problems in the interpretation of our results, we selected the control group with great care; we feel that the wives of

© Cancer Research Campaign 1997

the patient group could be safely compared with the wives of the controls – a similar percentage of married women was identified in the patient group as in the control group.

Although in our study, we have been unable to confirm a clear positive correlation between cancer of the penis among husbands and cancer of the cervix or vulva/vagina among their wives. We did find an increased (but non-significant) risk of preinvasive and invasive squamous cell carcinoma of the cervix. This association might be influenced by shared factors in marriage, such as smoking or dietary habits.

### REFERENCES

Beral V, Hermon C, Munoz N and Devesa SS (1994) Cervical cancer. *Cancer Surv* **19–20**: 265–285

Clark DP (1987) Condyloma acuminatum. *Dermatol Clin* **5**: 779–788

Drake M, Medley G and Mitchell H (1987) Cytologic detection of human papillomavirus infection. *Obstet Gynecol Clin North Am* **14**: 431–450

Graham S, Priore R, Graham M, Browne R, Burnett W and West D (1979) Genital cancer in wives of penile cancer patients. *Cancer* **44**: 1870–1874

Hunter RD (1995) Carcinoma of the cervix. In *Oxford Textbook of Oncology*, Peckham M, Pinedo HM and Veronesi U. (eds), pp. 1324–1348. Oxford University Press: Oxford

Iversen T (1981) Squamous cell carcinoma of the vulva. Localization of the primary tumor and lymph node metastases. *Acta Obstet Gynecol Scand* **60**: 211–214

Kjær SK, Van Den Brule AJC, Bock JE, Poll PA, Engholm G, Sherman ME, Walboomers JMM and Meijer CJLM (1996) Human papillomavirus – the most significant risk determinant of cervical intraepithelial neoplasia. *Int J Cancer* **65**: 601–606

Maiche AG and Pyrhonen S (1990) Risk of cervical cancer among wives of men with carcinoma of the penis. *Acta Oncologica* **29**: 569–571

Martinez I (1969) Relationship of squamous cell carcinoma of the cervix uteri to squamous cell carcinoma of the penis among Puerto Rican women married to men with penile carcinoma. *Cancer* **24**: 777–780

Oriel JD (1990) Identification of people at high risk of genital HPV infections. *Scand J Infect Dis Suppl* **69**: 169–172

Pizzocaro G, Piva L and Tana S (1995) Tumours of the penis. In *Oxford Textbook of Oncology*, Peckham M, Pinedo HM and Veronesi U. (eds). pp. 1450–1458. Oxford University Press: Oxford

Reddy CRRM, Gopal Rao T, Venkatarathnam G, Kameswari VR, Sashiprabha R and Raghavaiah NV (1977) A study of 80 patients with penile carcinoma combined with cervical biopsy study of their wives. *Int Surg* **62**: 549–553

Smith PG, Kinlen LJ, White GC, Adelstein AM and Fox AJ (1980) Mortality of wives of men dying with cancer of the penis. *Br J Cancer* **41**: 422–428

Waterhouse J, Muir C, Correa P and Powell J (eds) (1976). *Cancer Incidence in Five Continents*. International Agency for Research on Cancer: Lyon

© Cancer Research Campaign 1997